# Histopathological diagnosis in geriatric stomatology: a 45-year retrospective study from Brazil

**DOI:** 10.1590/1807-3107bor-2026.vol40.032

**Published:** 2026-06-12

**Authors:** Nayara Rubio Diniz del NERO, Anaíra Ribeiro Guedes Fonseca COSTA, Wender Rodrigues NAZÁRIO, Mikaelly Kuetrim Ribeiro BORGES, Marília Colucci PEREIRA, Gabriela Melo Terra PALAZZO, Débora de Oliveira SANTOS, Gabriel Tadeu COSTA JÚNIOR, Sérgio Vitorino CARDOSO, Marcelo José Barbosa SILVA, Adriano Mota LOYOLA, Paulo Rogério de FARIA

**Affiliations:** (a) Universidade Federal de Uberlândia – UFU, Department of Oral and Maxillofacial Pathology, Uberlândia, MG, Brazil.; (b) Private Practice, Catalão, Goiás, Brazil.; (c) Universidade Federal de Uberlândia – UFU, Institute of Biomedical Sciences, Department of Immunology, Uberlândia, Minas Gerais Brazil.

**Keywords:** Biopsy, Pathology, Oral, Oral Medicine, Geriatric Dentistry

## Abstract

This study analyzed the frequency and distribution of biopsied oral and maxillofacial lesions in a Brazilian geriatric population. Biopsy records of patients aged ≥60 years diagnosed over the last 45 years were retrospectively reviewed. Data on sex, age, anatomical site, and histopathological diagnosis were collected. Absolute and relative frequencies and their associations with clinicopathological variables were evaluated using the chi-square test and the two-proportions Z test. Among 21,367 lesions diagnosed between 1978 and 2023, 2,794 (13.1%) occurred in patients aged ≥60 years, predominantly in the 60–79 age group (90.6%) and in females (54.6%). The most frequent diagnostic categories were reactive lesions (56.6%), neoplasms (25.1%), and cysts/pseudocysts (8.5%). Fibrous hyperplasia (32.9%) and oral squamous cell carcinoma (15%) were the most common individual diagnoses. Females showed higher proportions of reactive lesions, immunological diseases, and bone lesions, with male-to-female ratios of 1:1.7, 1:2.2, and 1:5.7, respectively. The frequency and profile of oral and maxillofacial lesions in older Brazilians are consistent with previous reports. The high burden of inflammatory and neoplastic lesions reflects the cumulative effects of poor oral health and limited healthcare access. These findings highlight the need for strengthened preventive, diagnostic, and long-term oral healthcare strategies, particularly focusing on the aging population.

## Introduction

Global population aging has emerged as a major economic and healthcare challenge, driven by the rapid growth of older age groups worldwide. In 2019, individuals aged ≥ 65 years accounted for approximately 9% of the global population and are projected to reach 16% by 2050.^1^ Brazil, a country undergoing demographic transition, has followed a similar trend. The proportion of older adults increased from 4.9% in 1950 to 14.3% in 2020 and is expected to reach 32.2% by 2060.^2^

Increased life expectancy is accompanied by a higher risk of complex health conditions, including chronic diseases and functional disabilities, which are more prevalent in older adults than in younger populations.^3^ Oral health is a key determinant of quality of life in this age group and represents a substantial public health burden. Epidemiological data indicate that 57 to 77% of older adults are affected by oral lesions.^1,4^ Consequently, epidemiological studies are necessary to characterize the frequency and distribution of oral diseases in older individuals and to support public oral health policies aimed at promoting healthy aging.

Previous clinical studies^5-9^ and analyses of biopsy records^10-20^ have demonstrated a high frequency of denture-related conditions, malignant neoplasms, and odontogenic inflammatory lesions in geriatric populations. However, the incidence and prevalence of oral diseases are influenced by geographic, sociodemographic, cultural, and biological factors, as well as by temporal variables such as major global events (e.g., the COVID-19 pandemic) and periodic updates to the World Health Organization classification of tumors^21^. Long-term, region-specific studies are therefore critical for monitoring epidemiological trends and supporting evidence-based clinical practice. Within this context, this study aimed to analyze the frequency and distribution of oral and maxillofacial lesions diagnosed in a geriatric population from Brazil over an extended retrospective period.

## Methods

### Study design

This analytical cross-sectional study was conducted using a comprehensive archive of biopsy records. The study protocol was approved by the Institutional Ethics Committee on Human Research (90952425.1.0000.5152). All procedures complied with the principles of the World Medical Association’s Declaration of Helsinki^22^ and followed the Strengthening the Reporting of Observational Studies in Epidemiology (STROBE) guidelines.^23^ The Institutional Ethics Committee waived the requirement for informed consent due to the retrospective design and the use of anonymized data.

### Setting

The study sample consisted of biopsy specimens referred to the Department of Oral and Maxillofacial Pathology, School of Dentistry, Federal University of Uberlândia, Minas Gerais, Brazil, between March 1978 and September 2023. Data were retrospectively collected from archived biopsy files during the years 2023 and 2024.

### Participants

**Figure 1:**
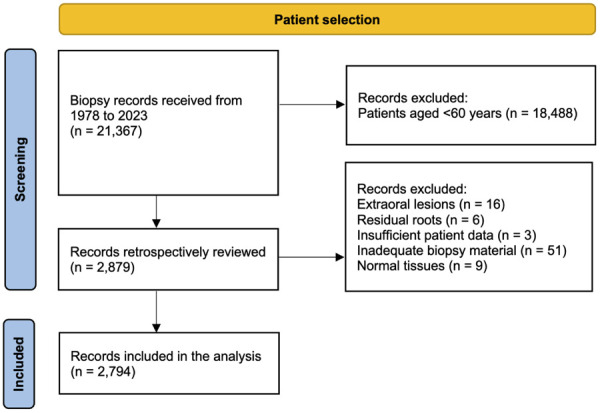
Study flowchart illustrating the inclusion and exclusion criteria for patient selection.

Eligible participants were patients aged ≥ 60 years who underwent biopsy of lesions located in the oral and maxillofacial region. The variables collected included age, sex, anatomical location of the lesion, and final histopathological diagnosis. Exclusion criteria comprised extraoral skin lesions, residual roots without associated periapical or periodontal pathology, cytopathological exams, biopsy records with missing demographic information (such as missing age or sex), and specimens reported as normal tissues. Figure 1 illustrates the sample selection process using a flowchart. Hematoxylin and eosin-stained slides from cases with inconclusive histopathological diagnoses or entities recently updated in classification were re-evaluated by two experienced pathologists (PRF and AML) according to the latest World Health Organization criteria.^21^

### Variables

For analytical purposes, older patients were stratified into two age groups: 60 to 79 years and ≥ 80 years. Anatomical locations were categorized as alveolar ridge, buccal mucosa, floor of mouth, gingiva, lips (upper and lower), lip commissures, lip frenulum, maxilla, mandible, oral vestibule, oropharynx, palate, salivary glands, tongue, teeth, maxillary sinuses, cervical lymph nodes, and multiple sites. Histopathological diagnoses were grouped into the following nine broad categories, adapted from the classification proposed by Cunha et al.:^20^ reactive and inflammatory lesions; benign and malignant neoplasms; cysts and pseudocysts; immunological and systemic diseases; oral potentially malignant disorders (OPMD); normal variations and tumor-like malformations; infectious diseases; pigmented and calcified lesions; and bone lesions.

### Statistical methods

Descriptive statistics included mean ± standard deviation for the continuous variable (age) and absolute and relative frequencies for categorical variables. Bivariate analyses were performed to assess associations between sex, age groups (60 to 79 years and ≥ 80 years), diagnostic categories, and decades of diagnosis using the Chi-square test and the two-proportions Z-test with continuity correction. To evaluate temporal trends and determine whether the relative frequency of each diagnostic category increased or decreased over time, the Cochran–Armitage test for trend was conducted, with decade treated as an ordinal variable. Results were reported with 95% confidence intervals. The significance level was set at 5%. Statistical analyses were performed using R (version 4.4.2, R Core Team 2021), with the summary tools and sjPlot packages.

### Study size

The study size was determined using a non-probability, convenience sampling approach that included all biopsy records available at the Institution until September 2023, resulting in 21,367 histopathological diagnoses.

## Results

### Participants

Among the 21,367 lesions diagnosed between 1978 and 2023, 2,794 (13%) involved patients aged ≥ 60 years.

### Descriptive data

**Figure 2:**
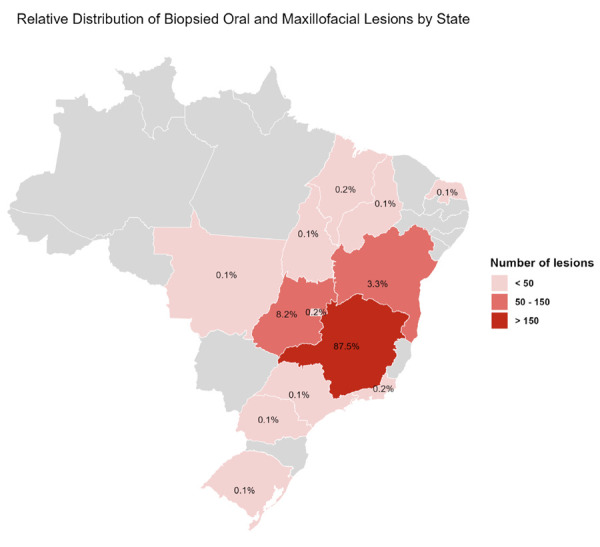
Distribution of biopsied oral and maxillofacial lesions among older patients across the states of Brazil.

Table 1 summarizes the absolute and relative frequencies of oral and maxillofacial lesions diagnosed in older patients, stratified by clinical variables. The mean age of the study population was 68.8 ± 7.2 years. Most patients (90.6%) belonged to the 60 to 79 year age group. A slightly higher proportion of biopsies was obtained from female patients (54.6%) than from male patients (45.4%), corresponding to a male-to-female ratio of 1:1.2. Approximately 40% of the specimens were referred through the Brazilian public health services, whereas 33% originated from private dental offices or academic institutions. In the remaining 26% of cases, the referral source could not be determined because biopsy records contained missing or illegible information or because some clinicians were affiliated with both public and private academic institutions. The attending patients came from 113 municipalities across 13 Brazilian states, although most were from Uberlândia (36%) and Minas Gerais State (52.5%) (Figure 2).

**Table 1:** Frequency distribution of oral and maxillofacial lesions in older patients according to clinical and demographic variables.

Clinical variables	n	%
Age (years)		
60–79	2,532	90.6
≥ 80	262	9.4
Sex		
Male	1268	45.4
Female	1526	54.6
Referring service		
Public health service	1129	40.4
Private health service	938	33.6
Not informed	727	26.0
Lesion type		
Mucosal	2323	83.1
Intraosseous	471	16.9
Location		
Alveolar ridge	490	17.56
Buccal mucosa	356	12.73
Tongue	336	12.02
Palate	281	10.05
Mandible	280	10.01
Upper lip	212	7.58
Maxilla	182	6.51
Floor of mouth	138	4.94
Alveolar mucosa	113	4.04
Gingiva	102	3.65
Lower lip	73	2.61
Salivary gland	32	1.14
Oropharynx	19	0.68
Maxillary sinuses	12	0.43
Multiple locations	10	0.36
Lip commissures	8	0.29
Teeth	2	0.07
Lip frenulum	1	0.04
Not informed	147	5.26

n: absolute frequency; %: relative frequency.

### Main results

Most biopsies performed in older patients involved oral mucosal lesions (83.1%), with the alveolar ridge being the most frequently affected site (17.6%). Intraosseous lesions were slightly more frequent in the mandible (10%) than in the maxilla (6.5%). Less than 1% of the lesions affected multiple sites. Among the nine histopathological categories evaluated, reactive and inflammatory lesions were the most frequent (56%), followed by benign and malignant neoplasms (25.1%), and cysts and pseudocysts of the oral and maxillofacial complex (8.5%).

Tables 2 and 3 summarize the distribution of diagnostic categories according to sex and age. Reactive lesions, immunological diseases, and bone lesions were significantly more frequent in females than in males, while the opposite trend was observed for neoplasms and cysts. When stratified by age, individuals aged 60 to 79 years showed a higher frequency of reactive lesions, neoplasms, and cysts compared with those aged ≥ 80 years. The most frequent diagnosis among females was fibrous hyperplasia (42.7%), whereas oral squamous cell carcinoma (OSCC) was the most common lesion in male patients (21.7%).

**Table 2:** Difference in the proportions of diagnostic categories according to sex in older patients with oral and maxillofacial lesions.

Diagnostic category	n (%)			M:F	p-value	95%CI	X
	Total	Male	Female				
Reactive and inflammatory lesions	1,584 (56.6)	584 (36.9)	1,000 (63.1)	1:1.7	< 0.001*	0.186 – 0.260	139.12
Benign and malignant neoplasms	701 (25.1)	426 (60.8)	275 (39.2)	1.6:1	< 0.001*	-0.189 – -0.123	89.13
Cysts and pseudocysts	238 (8.5)	145 (60.9)	93 (39.1)	1.6:1	< 0.001*	-0.075 – -0.031	24.66
Immunological and systemic diseases	76 (2.7)	24 (31.6)	52 (68.4)	1:2.2	0.020*	0.003 – 0.028	5.45
OPMD	62 (2.2)	32 (51.6)	30 (48.4)	1.1:1	0.386	-0.017 – 0.006	0.75
Normal variations and tumor-like malformations	53 (1.9)	24 (45.3)	29 (54.7)	1:1.2	1	-0.010 – 0.010	<0.01
Infectious diseases	30 (1.1)	19 (63.3)	11 (36.7)	1.7:1	0.072	-0.016 – 0.001	3.24
Pigmented and calcified lesions	30 (1.1)	11 (36.7)	19 (63.3)	1:1.7	0.435	-0.004 – 0.012	0.61
Non-neoplastic bone lesions	20 (0.2)	3 (15.0)	17 (85.0)	1:5.7	0.008*	0.002 – 0.015	6.33
Total	2794 (100)	1268 (45.4)	1526 (54.6)	1:1.2	-	-	-

n: absolute frequency; %: relative frequency; M:F: male to female ratio; OPMD: oral potentially malignant disorders; X: chi-square value; Two-proportions Z test with continuity correction, p < 0.05. *statistically significant.

**Table 3:** Differences in the proportions of diagnostic categories according to age group in older patients with oral and maxillofacial lesions.

Diagnostic category	n (%)			p-value	95%CI	X
	Total	60–79 years	≥ 80 years			
Reactive and inflammatory lesions	1,584 (56.6)	1,464 (57.8)	1,000 (63.1)	< 0.001*	0.055 – 0.185	13.43
Benign and malignant neoplasms	701 (25.1)	595 (23.5)	275 (39.2)	< 0.001*	-0.233 – -0.105	35.28
Cysts and pseudocysts	238 (8.5)	226 (8.9)	93 (39.1)	0.023*	0.014 – 0.073	5.20
Immunological and systemic diseases	76 (2.7)	73 (2.9)	52 (68.4)	0.148	0.001 – 0.034	2.09
OPMD	62 (2.2)	55 (2.2)	30 (48.4)	0.762	-0.027 – 0.017	0.09
Normal variations and tumor-like malformations	53 (1.9)	48 (1.9)	29 (54.7)	1.000	-0.018 – 0.017	<0.001
Infectious diseases	30 (1.1)	29 (1.1)	11 (36.7)	0.409	-0.003 – 0.018	0.68
Pigmented and calcified lesions	30 (1.1)	23 (0.9)	19 (63.3)	0.598	-0.005 – 0.016	0.28
Non-neoplastic bone lesions	20 (0.2)	19 (0.8)	17 (85.0)	0.773	-0.007 – 0.014	0.08
Total	2,794 (100)	2,532 (90.6)	262 (9.4)	-	-	-

n: absolute frequency; %: relative frequency; OPMD: oral potentially malignant disorders; X: chi-square value; Two-proportions Z test with continuity correction, p < 0.05. *statistically significant.

Table 4 shows the anatomical sites most frequently affected within each diagnostic category. Reactive and inflammatory lesions most commonly involved the alveolar ridge (22% of cases). The tongue was predominantly affected by benign and malignant neoplasms and infectious diseases, accounting for 18.8% and 28.3% of cases, respectively. Cysts and pseudocysts and non-neoplastic bone lesions were most frequently located in the mandible (53.4% and 55% of cases, respectively), whereas immunological and systemic diseases and OPMD mainly affected the buccal mucosa (50% and 25.8% of cases, respectively). Table 5 lists the 12 most frequent individual diagnoses in older patients, which together accounted for 71% (1,996 lesions) of all cases retrieved from the archives. The five most common diagnoses were fibrous hyperplasia (32.9%), OSCC (15%), mucositis (5%), radicular cyst (4.2%), and squamous papilloma (2.4%).

**Table 4:** Most frequently affected anatomical sites according to diagnostic category.

Diagnostic category	Total	Anatomical sites	n (%)
	n (%)		
Reactive and inflammatory lesions	1,584 (56.6)	Alveolar ridge	348 (22.0)
Benign and malignant neoplasms	701 (25.1)	Tongue	132 (18.8)
Cysts and pseudocysts	238 (8.5)	Mandible	127 (53.4)
Immunological and systemic diseases	76 (2.7)	Buccal mucosa	38 (50.0)
OPMD	62 (2.2)	Buccal mucosa	16 (25.8)
Normal variations and tumor-like malformations	53 (1.9)	Lower lip	15 (28.3)
Infectious diseases	30 (1.1)	Tongue	8 (26.7)
Pigmented and calcified lesions	30 (1.1)	Alveolar ridge and floor of mouth	12 (40.0)
Non-neoplastic bone lesions	20 (0.2)	Mandible	11 (55.0)
Total	2794 (100)	-	-

OPMD = oral potentially malignant disorders; n = absolute frequency; % = relative frequency.

**Table 5:** Twelve most frequent oral and maxillofacial lesions in older patients and their distribution according to sex and age groups.

Diagnostic category	Frequency n (%)				
	Total	Sex		Age	
		Male	Female	60–79 years	≥ 80 years
Fibrous hyperplasia	921 (32.9)	269 (29.2)	652 (70.8)	867 (94.1)	54 (5.9)
Oral squamous cell carcinoma	420 (15.0)	276 (65.7)	144 (34.3)	357 (85.0)	63 (15.0)
Unspecified mucositis	141 (5.0)	68 (48.2)	73 (51.8)	123 (87.2)	18 (12.8)
Radicular cyst	118 (4.2)	54 (45.8)	44 (37.3)	113 (95.8)	5 (4.2)
Squamous papilloma	66 (2.4)	35 (53.0)	31 (47.0)	60 (90.9)	6 (9.1)
Periapical granuloma	65 (2.3)	31 (47.7)	34 (52.3)	61 (93.8)	4 (6.2)
Pyogenic granuloma	63 (2.2)	32 (50.8)	31 (49.2)	54 (85.7)	9 (14.3)
Epithelial hyperplasia and hyperkeratosis	57 (2.0)	28 (49.1)	29 (50.9)	52 (91.2)	5 (8.8)
Oral lichen planus	40 (1.4)	13 (32.5)	27 (67.5)	39 (97.5)	1 (2.5)
Mucus extravasation phenomenon	38 (1.3)	16 (42.1)	22 (57.9)	35 (92.1)	3 (7.9)
Oral leukoplakia	35 (1.2)	20 (57.1)	15 (42.9)	32 (91.4)	3 (8.6)
Unspecified odontogenic cyst	32 (1.1)	23 (71.9)	9 (28.1)	30 (93.8)	2 (6.3)

n: absolute frequency; %: relative frequency.

Considering the histopathological categories used to classify the oral and maxillofacial lesions, the most frequent entities within each category were as follows: fibrous hyperplasia (58.1% of the reactive and inflammatory lesions), OSCC (59.8% of benign and malignant neoplasms), radicular cyst (49.6% of cysts and pseudocysts), oral lichen planus (52.6% of immunological diseases), oral leukoplakia (56.4% of OPMD), hemangioma (41.5% of tumor-like malformations), oral candidiasis (33.3% of infectious diseases), sialolithiasis (36.7% of pigmented and calcified lesions), and benign fibro-osseous lesions (60% of bone lesions).

Among neoplastic lesions, malignant and benign neoplasms accounted for 74.2% and 25.8%, respectively. Malignant epithelial tumors were the most prevalent (66.6%), followed by benign epithelial tumors (9.8%). Benign mesenchymal tumors accounted for 6.7% of all neoplasms, with lipoma being the most frequent entity (3.8%). Salivary gland tumors comprised 10.0% of all neoplasms, including benign (5.4%) and malignant (4.6%) lesions. Pleomorphic adenoma was the most common benign salivary gland tumor (3.4%), and adenocarcinoma not otherwise specified was the most frequent malignant salivary gland neoplasm (1.3%). Odontogenic tumors represented 3.7% of neoplastic lesions, with ameloblastoma being the most prevalent diagnosis (2.8%). Hematolymphoid tumors accounted for 3.1% of neoplasms, among which large B-cell lymphoma was the most frequent subtype (1.1%). Other malignancies, including mesenchymal, odontogenic, and metastatic tumors, represented less than 0.5% of all neoplasms affecting the oral and maxillofacial region.

### Other analyses

**Figure 3:**
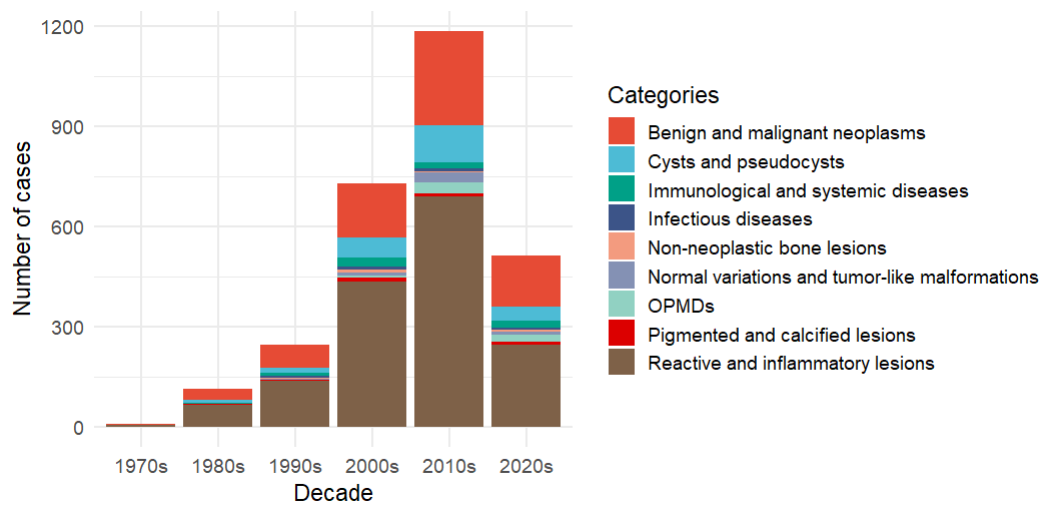
Distribution of biopsied oral and maxillofacial lesions among older patients across decades according to diagnostic categories.

The Cochran–Armitage test revealed a significant increasing trend in OPMDs over the study period (Z = 4.75, p < 0.001), whereas the frequency of reactive and inflammatory lesions showed a significant decreasing trend over the decades (Z = −2.89, p = 0.004). Notably, the number of reactive and inflammatory lesions declined from 691 cases in the 2010s to 246 cases in the 2020s. In addition, the two-proportions Z test demonstrated a significant difference in the proportion of reactive and inflammatory lesions between the 2010s and 2020s (X = 15.2, 95% CI = 0.05–0.15, p < 0.001). The distribution of diagnostic categories over the decades is illustrated in Figure 3.

## Discussion

In this 45-year retrospective analysis, lesions diagnosed in patients aged ≥ 60 years accounted for 13.1% of all oral and maxillofacial biopsies registered at our institution. This proportion is consistent with previous biopsy-based studies, in which older adults represented between 9.2% and 24.1% of all histopathological diagnoses reported worldwide.^11-20^ One major source of heterogeneity in the literature is the definition of older people. Previous investigations have adopted different age thresholds, including ≥ 50 years,^17^ ≥ 60 years,^10,12,14,15,18-20^ and ≥ 65 years.^11,13,16^ These differences likely reflect variations in life expectancy, demographic structure, and sociocultural characteristics among Finland,^10^ England,^11^ Brazil,^12,18-20^ the United States,^13,18^ Taiwan,^15^ South Korea, Japan, Iran, Canada,^16^ and India,^1^, countries where such epidemiological investigations were conducted.

The frequency of biopsies in older people peaked in the 60–79 year age range, consistent with findings from other studies that analyzed the same age range^12,14,15,18,20^. A higher proportion of biopsies in female patients was reported in seven^11-14,16,19,20^ of the eleven surveys, with male-to-female ratios ranging from 1:1.2 to 1:2. In contrast, only three studies conducted in Taiwan,^15^ India,^17^ and Brazil^18^ reported a higher number of biopsies among males. This difference might be associated with geographic, sociodemographic, and cultural factors^18,20^ specific to each country, including the relative proportions of men and women in the population and structural gender inequalities in education, income, and access to healthcare. Although women often experience social and economic disadvantages, evidence indicates that men are more likely to neglect oral health, maintain poorer hygiene habits, and seek dental care less frequently than women. Men also tend to access dental care primarily for acute conditions or emergencies rather than for preventive care.^24^ Consequently, men show a higher prevalence of periodontal and periapical diseases, as well as oral cancer associated with tobacco use, alcohol consumption, and chronic sun exposure.^24^ These behavioral patterns may explain the slightly higher proportion of oral neoplasms and cysts observed among males compared with females in the present study, with a male-to-female ratio of 1.6:1.

Previous studies have identified the buccal mucosa,^16-18^ gingiva,^1^, alveolar ridge,^14^ and tongue^20^ as the most frequently biopsied anatomical sites in older adults, with intraosseous lesions reported more frequently in the mandible than in the maxilla.^14^ Our findings are consistent with the literature, as the alveolar ridge and mandible were the most commonly affected sites for oral mucosal and intraosseous lesions, respectively. Oral mucosal lesions in the alveolar ridge of older people are probably associated with prosthetic denture use.^8^ In Brazil, nearly 80% of the geriatric population has been reported to be edentulous.^25^ Regarding intraosseous lesions, the relative predominance of mandibular involvement reflects the higher incidence of odontogenic cysts and tumors in this bone. This pattern has been attributed to the greater frequency of impacted teeth in the mandible compared with the maxilla,^26^ as well as to potentially higher burden of entrapped odontogenic remnants in mandibular bone.^27^

Reactive and inflammatory lesions were the most frequent diagnoses in our study, with fibrous hyperplasia representing the predominant one, followed by benign and malignant neoplasms. Within the neoplastic category, OSCC was the most common diagnosis. These findings are consistent with previous reports showing that non-neoplastic proliferative lesions account for 32.4%–66.1% of histopathological diagnoses in older populations.^11-16,18,20^ OSCC has likewise been reported as the most frequent diagnosis among older patients in several studies,^15-17,19^ comprising between 12.2% and 34% of all biopsies. In contrast, a Finnish survey identified radicular cysts as the most common lesion, accounting for 18% of biopsy samples.^10^. Despite the high frequency of malignant epithelial lesions, OPMDs accounted for only 2% of biopsies in the present study, a proportion substantially lower than that reported in other Brazilian studies, such as those by Cunha et al. (10%)^20^ and Silva et al. (9.9%).^18^ This discrepancy may reflect variations in oral cancer epidemiology within Brazil. Although the national incidence of oral cancer is estimated at approximately 4.9 per 100,000 people, the southeastern region shows the highest incidence (6.3 per 100,000 population).^28^ Such regional differences likely mirror risk factor exposure, including tobacco and alcohol use, as well as disparities in access to and effectiveness of local cancer screening programs. Given the impact of delayed diagnosis on patient survival and quality of life, these findings underscore the need for strengthened public health strategies aimed at improving surveillance of OPMDs and promoting early diagnosis of OSCC in older populations.^20^

It is important to note that the diagnosis of OPMDs requires clinicopathological correlation, as several histopathological features overlap with those of non-neoplastic conditions that must be excluded during anamnesis and oral examination.^29^ While histopathology is indispensable for grading epithelial dysplasia and detecting occult OSCC, it is not diagnostically definitive when interpreted in isolation. Consequently, the frequency of OPMDs in the present study may be underestimated, owing to incomplete clinical information or the absence of diagnostic hypotheses in biopsy records, limiting the pathologist’s role in the workflow for OPMD diagnosis.

Our results indicate a higher frequency of reactive lesions, immunological diseases, and non-neoplastic bone lesions in females than in males, with male-to-female ratios of 1:1.7, 1:2.2, and 1:5.7, respectively. These results are consistent with other Brazilian studies reporting a female predominance in reactive lesions,^30^ immunological diseases,^31^ and benign fibro-osseous lesions.^32^ However, the underlying reasons for this epidemiological pattern remain insufficiently explored beyond cultural influences. Data from the southern region of Brazil have shown that reactive hyperplastic lesions of the oral cavity occur more frequently in females, except for peripheral giant cell lesions.^30^ Similarly, an investigation of immune-mediated diseases in the same region reported that females accounted for 73.7% of the affected individuals, with a mean age of 60.2 years.^31^ In the northeast region of Brazil, a comparable female predominance has been described for cemento-ossifying fibroma and other fibro-osseous lesions, in which females accounted for 75.6% of cases, corresponding to a male-to-female ratio of 1:3.1.^32^

Although the clinical, radiographic, and histopathological characteristics of these lesions are well documented, the potential etiopathogenetic mechanisms underlying their higher prevalence in females are rarely discussed in the literature. Reactive and immunological diseases share common immunoinflammatory pathogenesis, although their initiating etiological factors differ. Sex-related differences in inflammatory and immune responses may partly explain the increased susceptibility of females to these conditions, particularly in older age groups. Older women may exhibit a more pronounced chronic low-grade pro-inflammatory state, characterized by increased numbers of natural killer cells, CD4+ T cells, B cells, higher CD4/CD8 ratios, and elevated levels of IL-10 and immunoglobulins compared with men.^33^

Regarding benign fibro-osseous lesions, which showed the most pronounced male-to-female disparity, the involvement of sex hormones in bone physiology has been proposed as a contributing factor. Estrogen, in particular, plays a central role in skeletal homeostasis. In post-menopausal women, low estrogen levels may be associated with decreased expression of osteoprotegerin and increased levels of receptor activator of nuclear factor kappa-B and tumor necrosis factor α, leading to enhanced bone resorption.^34^ G protein-coupled estrogen receptors may also modulate mesenchymal cell proliferation and differentiation, thereby contributing to the development of fibro-osseous lesions. Although mutations in the gene encoding the G-protein alpha subunit that characterize fibrous dysplasia are not sex-linked, the constitutive activation of adenylyl cyclase and the consequent elevation of cyclic adenosine monophosphate levels may activate intracellular signaling pathways mediated by estrogen receptors.^35^ These mechanisms provide a plausible biological basis for the marked female predominance observed in benign fibro-osseous lesions in older patients.

Regardless of sex, reactive and inflammatory lesions constituted the most frequent diagnostic category in this study, followed by neoplasms and jaw cysts. Excluding neoplastic lesions, fibrous hyperplasia and radicular cysts were the most common diagnoses within their respective categories. The high frequency of these lesions in the Brazilian population reflects longstanding deficiencies in oral health status and limited access to oral health services. National survey data indicate that only 7.3% of individuals aged 65 to 74 years did not require prosthodontic dental care.^36^ Fibrous hyperplasia typically arises after chronic mucosal trauma, most often related to ill-fitting or long-term use of dentures. Such conditions are frequently associated with financial constraints and difficulties in accessing or adapting to new prostheses.^37^ It is also important to consider the historical context of the present findings. The lesions analyzed reflect oral health conditions prevailing several decades earlier, largely before 1965. In 1980, for instance, dental caries were highly prevalent in Brazil, with a national DMFT (Decayed, Missing, and Filled Teeth) index of 7.3.^38^ In this scenario, a high burden of periapical lesions, inflammatory cysts, and reactive mucosal lesions would be expected.

Among neoplastic lesions, squamous papilloma (9.4%) ranked second in frequency, followed by squamous cell carcinoma (59.85%). Squamous papilloma is a common benign epithelial tumor of the head and neck. Although it was long considered to be driven by human papillomavirus (HPV) infection, more recent studies have shown that the prevalence of HPV in benign oropharyngeal tumors is below 15%.^39,40^ Squamous papilloma has also been reported as one of the most frequent histopathological diagnoses in the pediatric population previously investigated by our research group.^41^ Other benign neoplasms identified in the geriatric population included lipoma, pleomorphic adenoma, and ameloblastoma, accounting for up to 4% of this diagnostic category. The frequency of these tumors was lower than that reported in other Brazilian surveys.^12,18,20^ This discrepancy may be partly explained by referral patterns, as patients with these conditions may be more frequently directed to medical rather than dental services for surgical management, especially in the context of the private healthcare service.

Infectious lesions were uncommon in the studied population and were mainly represented by oral candidiasis, which is also associated with inadequate use or hygiene of dental prostheses. It should be noted that Candida spp. infections of the oral mucosa are typically underrepresented in biopsy-based surveys when compared with clinical and epidemiological studies,^6,8^ as diagnosis is often based on clinical evaluation and cytopathological tests rather than histopathological biopsy. A similar explanation applies to normal variations and tumor-like malformations, such as hemangiomas and varicosities.

Time-trend analysis demonstrated that both the absolute number and the relative proportion of reactive and inflammatory lesions declined over the study period, especially between the 2010s and 2020s. This pattern could be attributed to the impact of the COVID-19 pandemic on the diagnosis of oral diseases at the beginning of the 2020s. A Brazilian study reported a 75.6% reduction in biopsy procedures in the southern region of Brazil during this period.^42^ Although the apparently stable number of neoplastic diagnoses in the 2020s suggests a prioritization of lesions with clinical suspicion of malignancy over traumatic or inflammatory conditions, the possibility that opportunities for early diagnosis of oral cancer were missed cannot be excluded. Conversely, the observed increase in OPMD diagnoses may reflect heightened awareness of these lesions by the dental community in recent years, driven by greater emphasis in both clinical practice and scientific research over the past decade.

Finally, some limitations of this study should be acknowledged to facilitate a proper interpretation of the descriptive results. First, this was a biopsy-based survey of oral and maxillofacial lesions affecting the older population, thereby excluding lesions diagnosed solely on clinical settings. Therefore, the data do not allow estimation of true incidence or prevalence rates. The use of convenience sampling and the inclusion of only individuals who underwent oral biopsy and histopathological examination further limit the representativeness of the sample. Besides, the consistency of the results is dependent on the completeness and accuracy of retrospectively collected records. Relevant demographic and clinical data, such as lesion location and referral source, were missing in a proportion of cases. Incomplete paper-based and electronic dental records remain a recognized challenge in dental practice because of several factors, including high clinical workload, time constraints, and competing tasks for health providers.^43^ Nevertheless, long-term observational studies provide valuable epidemiological insights.

## Conclusions

The frequency and distribution of oral and maxillofacial lesions among older Brazilians are consistent with those reported in comparable investigations. The high burden of inflammatory and neoplastic lesions highlights the need for oral health services tailored to individuals aged ≥ 60 years, with emphasis on prevention, early detection, personalized treatment, and long-term follow-up. Strengthening coordinated actions within both public and private healthcare systems is essential to improve oral health outcomes and quality of life in the aging population.

## Data Availability

The datasets generated during and/or analyzed during the current study are available from the corresponding author on reasonable request.
